# Physical Health and Cognitive Function Independently Contributed to Functional Disability among Chinese Older Adults: Data from Two Asian Metropolises

**DOI:** 10.4061/2011/960848

**Published:** 2011-09-14

**Authors:** Lei Feng, Tze-Pin Ng, Yanling He, Chunbo Li, Ee-Heok Kua, Mingyuan Zhang

**Affiliations:** ^1^Gerontological Research Programme, Yong Loo Lin School of Medicine, National University of Singapore, Singapore; ^2^Department of Psychological Medicine, Yong Loo Lin School of Medicine, National University of Singapore, Singapore; ^3^Shanghai Mental Health Centre, Shanghai Jiaotong University, Shanghai 200030, China

## Abstract

*Objective*. We aimed to examine the independent contributions of physical health and cognitive function to disability among Chinese older adults living in two Asian metropolises and explore the potential influences of environment. *Design and Participants*. Cross-sectional analysis based on data from two population-based studies: the Shanghai Survey of Alzheimer's Disease and Dementia (*n* = 4639) and the Singapore Longitudinal Ageing Study (*n* = 2397). Disability was defined as needing help in at least one activity of daily living. *Results*. The prevalence of functional disability was higher in Shanghai sample (5%) than that in Singapore sample (1.8%). Number of chronic diseases, self-rated health status, cognitive function (measured by the Mini-Mental State Examination), and environment (Singapore versus Shanghai) significantly contributed to functional disability independent of each other. The adjusted Odds Ratio was 1.35 (95%CI 1.22–1.50), 2.85 (95% CI 2.36–3.43), 0.89 (95% CI 0.85–0.94), and 0.68 (95% CI 0.48–0.96), respectively. The strength of associations between health variables and disability appeared to be influenced by environment. *Conclusion*. Physical health and cognitive function independently contributed to functional disability. The associations are modulated by environmental factors.

## 1. Introduction

Disability prevalence increases with advanced age [1], and disability in the elderly population is a major public health problem [2]. This is especially crucial for Chinese: there are 88 million persons aged 65 and above in China alone, and the number is projected to increase to 341 million in 2050 [1]; importantly, disability prevalence in Chinese elderly population has been increasing [3–5] in contrast to a declining trend in the developed countries [6, 7]. 

There is a big gap between the scale of the problem and the quantity and quality of available information. The first population-based report from China was published 16 years ago [8], and countable number of publications [3–5, 8–12] exist in the literature. 

In this study, with large population-based data from Shanghai (the biggest city in China) and Singapore (a city country in Southeast Asia, with majority of the residents being descendents of immigrants from South China), we aimed to examine the independent contributions of physical and cognitive health to disability in Chinese older adults and explore the potential influences of environment.

## 2. Methods

### 2.1. Participants

Participants of the present study were identified from two big population-based studies: the Shanghai Survey of Alzheimer's Disease and Dementia (SSADD) and the Singapore Longitudinal Ageing Study (SLAS). The SSADD was conducted in 1987 based on a probability sample of 5055 community-dwelling Chinese older adults from Jing'an district of Shanghai city. The SLAS was conducted among a total population sample of 2808 older adults from South East Region of Singapore. Baseline assessments of the SLAS were completed from September 2003 to December 2005. Details of the SSADD [8, 13] and SLAS [14] have been described elsewhere.

For the present analysis, we selected 4639 participants from the SSADD sample and 2397 participants from the SLAS cohort. All participants had complete data on demographic information, physical health, and cognitive function and obtained a Mini-Mental State Examination (MMSE) total score of at least 21 points. 

### 2.2. Measures

Face-to-face interviews were conducted by psychiatrists/psychiatric nurses (in Shanghai) or trained research nurses (in Singapore), and data were collected for a wide range of variables. For the present analysis, we extracted the following variables from the databases: age, sex, functional status, chronic diseases, self-rated health status, and MMSE total score. 

Functional status was assessed by the participant's level of dependency in performing 8 Activities of Daily Living (ADL): eating, grooming, dressing, transferring, walking, toileting, bathing, and climbing stairs. Disability was defined as needing help in at least one ADL.

A list of 32 medical conditions was included in the SSADD interview schedule. The participants were asked “Do you have or not have any of the following illnesses or conditions at the present time?" In the SLAS, a list of 14 medical conditions was covered in the interview. The participants were asked “Do you have a history of this medical problem?” Medical conditions that were not included in the list were recorded under “any other problems.” We selected ten chronic diseases on which data were available from both samples: hypertension, diabetes, heart diseases (in Singapore: defined as any of heart attack, heart failure, or atrial fibrillation), stroke (in Shanghai: effects of stroke), kidney disorder (in Singapore: kidney failure), chronic obstructive lung disease (in Shanghai: emphysema/bronchitis), asthma, arthritis (in Shanghai: arthritis or rheumatism), mental illness, and cancer (in Singapore, identified from “any other problems”). In statistical analysis, the number of chronic diseases was used as an objective measure of physical health. 

In Singapore, self-rated health status was assessed using a single question: “In general, would you say your health is: excellent, very good, good, fair, or poor?” In Shanghai, the same question was asked but there were four choices: excellent, good, fair, poor. We grouped “excellent” and “very good” together and created a new variable “self-rated health status”: 1 = excellent or very good, 2 = good, 3 = fair, and 4 = poor. This was used as a continuous variable in multivariate analyses (score ranged from 1 to 4, with higher value representing poorer health status). 

Cognitive function was assessed by Mini-Mental State Examination (MMSE) test [15]. The test has been validated in Shanghai [16] and Singapore [17], respectively. Summed scores of MMSE range from 0 to 30, higher values denoting better cognitive functioning.

### 2.3. Statistical Analysis

Chi-square test (for dichotomous variables) or independent sample *t* test (for continuous variables) was used to compare the difference regarding various characteristics between the two study samples. Multiple logistic regressions were used to examine the relationship between health factors and disability, and the influence from environment. In analysis based on the combined sample, age, sex, number of chronic diseases, self-rated health status, MMSE total score, and environment (Singapore versus Shanghai) were included in the regression model. The same set of variables (except environment) was used in the stratified analysis. All analyses were performed using SPSS version 18.0.

## 3. Results

Table 1 compared various characteristics between the two study samples. The prevalence of disability was much higher in Shanghai sample (5.0%) than that in the Singapore sample (1.8%). However, the Shanghai participants were also older, had more chronic diseases, had poorer self-rated health status, and obtained lower MMSE total score as compared to their Singapore counterparts (Table 1). A scrutiny of the eight ADL items revealed that the difference was only significant for bathing (3.5% versus 0.4%) and climbing stairs (3.1% versus 1.2%).

In multiple logistic model based on the combined sample, number of chronic diseases, self-rated health status, MMSE total score, and environment were significantly associated with disability (Table 2). The Odds Ratio was 1.35 (95% CI 1.22–1.50) for number of chronic diseases, 2.85 (95% CI 2.36–3.43) for self-rated health status (continuous), 0.89 (95% CI 0.85–0.94) for MMSE total score, and 0.68 (95% CI 0.48–0.96) for environment (Singapore versus Shanghai), respectively. 

Results from stratified analysis showed that the strength of associations between health variables and disability differed between the two samples (Table 3). For example, in the Shanghai sample, one point increase on the 4-point self-rated health status scale was associated with 3.13 times (95% CI 2.55–3.85) higher odds of having disability while the Odds Ratio was only 1.73 (95% CI 1.07–2.81) in the Singapore sample. For MMSE total score, the corresponding Odds Ratio was 0.91 (95% CI 0.86–0.96) in the Shanghai sample and 0.83 (95% CI 0.73–0.94) in the Singapore sample. 

## 4. Discussion

Based on data from two population-based studies, we found that the number of chronic diseases, self-rated health status, cognitive function, and unmeasured environment factors represented by study sample were significantly associated with functional disability among Chinese older adults.

The contributions of physical health [8, 18] and cognitive function [19, 20] to functional disability have been documented and were well replicated in our analysis. With large population-based data, we firstly quantified the strength of differential contributions of each of self-reported physical health, self-rated health status, and cognitive function to disability in Chinese elderly population. The findings are relevant and important for policymakers, medical practitioners, and academics. 

We found that environment was significantly associated with disability. Participants in the Singapore sample were 32% less likely to have disability compared with participants in the Shanghai sample. Participants from both studies were Chinese older adults living in big city, and a number of important covariates were adjusted in multiple regression models. It is less likely that the observed difference was caused by unmeasured factors at individual level. 

We did not collect data on objective indicators of environmental factors. However, a careful examination of the two most difficult ADL tasks (bathing and climbing stairs) provided us with plausible explanations. In 1987, most families in Shanghai had no bathroom and shower, and bathing was considered as a complicated task [8]. The stairs were small, narrow, and difficult to climb. In contrast, the majority of SLAS participants lived in public houses that have standard bathroom and shower and easy-to-climb stairs. The differences in immediate environment could be major cause of the observed differences. 

Our study provided fresh evidence on the role of environmental factors in disablement process [21–23]. As pointed out by Verbrugge and Jette: “Disability is not a personal characteristic, but is instead a gap between personal capability and environmental demand…disability can be diminished swiftly and markedly if the physical or mental demands of a given task are reduced” [22]. Our findings suggest that environment not only affects the prevalence of disability, but also modifies the strength of associations between disability and health variables. 

Strengths of our study include concurrent measuring of various health factors and simultaneous inclusion of those factors in multivariate model. This made a cross-comparison of the strength of independent associations possible. However, no causal relationship could be drawn given cross-sectional nature of the study design. 

In conclusion, physical health and cognitive function were significantly associated with disability among Chinese older adults living in Asian metropolises. The association could be influenced by environment. A comprehensive approach should be adopted in disability prevention.

##  Conflict of Interests

There are neither financial nor dual commitments that represent potential conflict of interests.

##  Authors' Contribution

All the authors contributed substantially to the design, analysis and interpretation of the data and participated in drafting or revising the paper.

## Figures and Tables

**Table 1:** Characteristics of the participants.

	Shanghai (*n* = 4639)	Singapore (*n* = 2397)	*P*
Age (years)	68.3 (7.8)	65.6 (7.2)	<0.001
Range (years)	55–93	55–97	
55–64	1167 (31.6%)	1158 (48.3%)	<0.001
65–74	2074 (44.7%)	938 (39.1%)	
75+	1098 (23.7%)	301 (12.6%)	
Female	2510 (54.1%)	1495 (62.4%)	<0.001
Number of chronic diseases	1.29 (1.18)	1.16 (1.09)	<0.001
Self-rated health status*	2.59 (0.77)	2.38 (0.65)	<0.001
Excellent or very good	164 (3.5%)	145 (6.0%)	<0.001
Good	2241 (48.3%)	1257 (52.4%)	
Fair	1569 (33.8%)	925 (38.6%)	
Poor	665 (14.3%)	70 (2.9%)	
MMSE total score	26.6 (2.8)	27.6 (2.2)	<0.001
Functional disability^†^ (whole sample)	233 (5.0%)	43 (1.8%)	<0.001
Within age group 55–64	15 (1.0%)	14 (1.2%)	0.65
Within age group 65–74	82 (4.0%)	18 (1.9%)	0.004
Within age group: 75+	136 (12.4%)	11 (3.7%)	<0.001
Disability in any of the ADL items			
Eating	11 (0.2%)	5 (0.2%)	0.81
Grooming	29 (0.6%)	8 (0.3%)	0.11
Dressing	38 (0.8%)	11 (0.5%)	0.09
Transferring	37 (0.8%)	9 (0.4%)	0.04
Walking	43 (0.9%)	14 (0.6%)	0.13
Toileting	38 (0.8%)	17 (0.7%)	0.62
Bathing	162 (3.5%)	9 (0.4%)	<0.001
Climbing stairs	144 (3.1%)	28 (1.2%)	<0.001

*Range from 1 (excellent or very good) to 4 (poor), with higher value representing poorer health.

^†^Defined as needing assistance in performing at least one of the eight Activities of Daily Living.

Data are mean (SD) or number (%).

MMSE: Mini-Mental State Examination.

**Table 2:** The independent contributions of health variables and environment to disability*: combined analysis.

Variable	Logistic regression	
	OR (95% CI)	*P*
Number of chronic diseases	1.35 (1.22–1.50)	<0.001
Self-rated health status^†^	2.85 (2.36–3.43)	<0.001
MMSE total score	0.89 (0.85–0.94)	<0.001
Environment		
Shanghai 1987	1.0	
Singapore 2003	0.68 (0.48–0.96)	0.03

*Defined as needing assistance in performing at least one of the eight Activities of Daily Living.

^†^Range from 1 (excellent or very good) to 4 (poor), with higher value representing poorer health.

MMSE: Mini-Mental State Examination.

Variables in the model: age, gender, number of diseases, self-rated health status, MMSE total score, and environment (study sample).

**Table 3:** Association between health factors and disability in Shanghai and Singapore: stratified analysis.

Variable	Shanghai (*n* = 4639)	Singapore (*n* = 2397)
	OR (95% CI)	OR (95% CI)
Number of chronic diseases	1.36 (1.22–1.52)	1.54 (1.21–1.97)
Self rated overall health	3.13 (2.55–3.85)	1.73 (1.07–2.81)
MMSE total score	0.91 (0.86–0.96)	0.83 (0.73–0.94)

Variables in the model: age, gender, number of chronic diseases, self-rated health status, and MMSE total score.
